# Failed transtibial pullout repair of the medial meniscus posterior root

**DOI:** 10.1016/j.radcr.2023.01.006

**Published:** 2023-02-03

**Authors:** Gabrielle Wasilewski, Hamidou Drammeh, Mohamed Belal, Zuhaib Khokhar, Andrew Pasion, Emad Allam

**Affiliations:** Loyola University Chicago and Loyola University Medical Center, 2160 S 1st Ave, Maywood, IL 60153, USA

**Keywords:** Medial meniscus, Meniscus tear, Meniscus root tear, Meniscus root repair, Transtibial pullout repair

## Abstract

Transtibial pullout repair is increasingly being utilized for repair of meniscal root tears. Loosening and suture pullout may occur after such a repair but has not been reported in the radiology literature. We present a case of a transtibial pullout repair complicated by suture pullout, recognized on MRI and confirmed on subsequent arthroscopy. This complication may occur in the immediate postoperative period and is important to recognize as it requires surgical management.

## Case presentation

A 51-year-old female presented with acute onset right knee pain which occurred while performing squat exercises. She felt a pop in the posterior aspect of the knee at the time of the injury. On physical exam, there was pain primarily along the posteromedial aspect of the knee and there was a palpable Baker's cyst. There was no ligamentous instability.

She had no relevant past medical history. She worked in the local fire department.

**Fig. 1:**
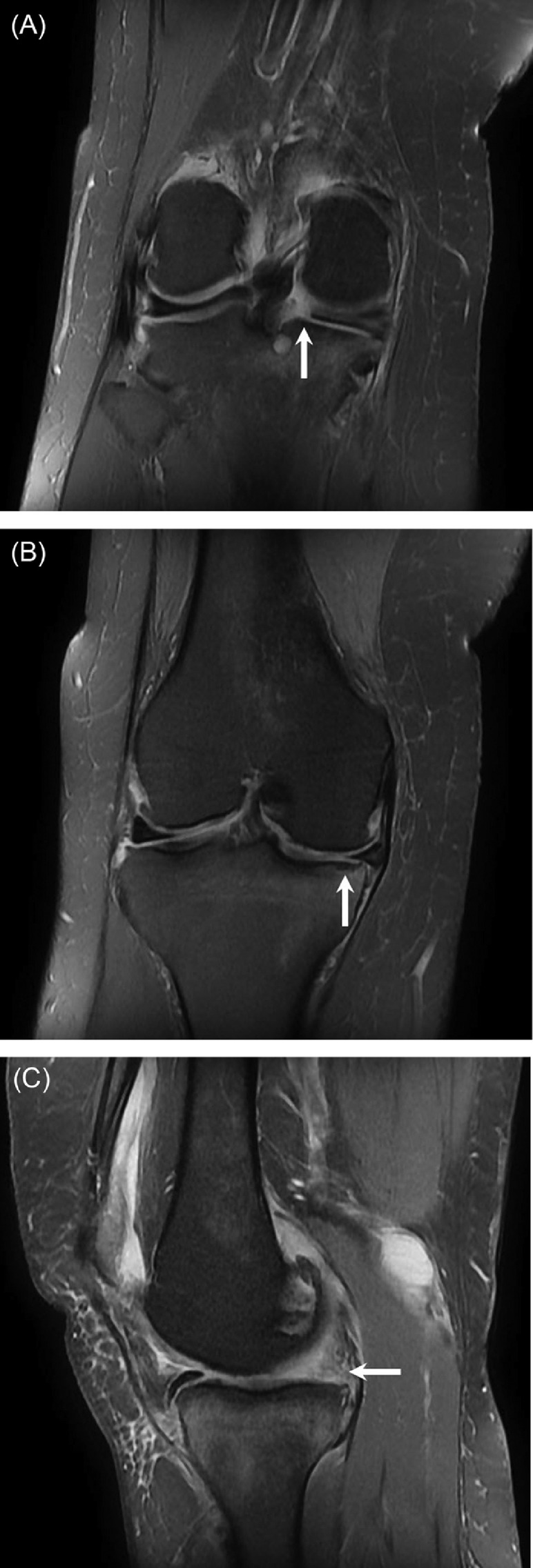
(A) Preoperative MRI. Coronal PD FS MRI image shows a complete radial tear (arrow) of the posterior root of the medial meniscus. (B) Preoperative MRI. Coronal PD FS MRI image shows extrusion of the body of the medial meniscus from the joint space. Partial thickness chondrosis is visible in the medial femoral condyle. There is subchondral marrow edema in the medial tibial plateau with a small area of linear hypointensity suggestive of subchondral insufficiency fracture (arrow). (C) Preoperative MRI. Sagittal PD FS MRI image shows complete absence of the posterior root of the medial meniscus, compatible with a complete root tear (arrow). There is partial thickness chondrosis in the medial femoral condyle, subchondral marrow edema in the medial tibial plateau, partially imaged knee joint effusion and Baker's cyst.

MRI at that time showed a complete tear of the medial meniscus posterior root (Fig. 1). Partial thickness chondrosis was seen in the medial femoral condyle. There was subchondral marrow edema in the medial tibial plateau and a subtle subchondral insufficiency fracture.

Arthroscopy performed approximately 7 weeks after the MRI demonstrated a complete tear of the medial meniscus posterior root. The posterior root footprint and meniscus tissue were debrided with a shaver. A socket was created in the proximal tibia using a drill and a wire loop was passed through the tibia. Two fiberwire sutures were passed through the posterior root meniscus tissue in a luggage-tag configuration. The sutures were then loaded through the wire loop and pulled through the tibial socket and tunnel. The sutures were loaded into a suture anchor. The anchor was then drilled and inserted into the proximal tibia. The meniscus was noted to be appropriately docked into the tibial socket and was stable on probing. There was grade II chondrosis in the medial femoral condyle, intact cartilage in the medial tibial plateau, grade III chondrosis in the femoral trochlea, and grade IV chondrosis in the patella. Chondroplasty of the medial femoral condyle and femoral trochlea was performed. The cruciate ligaments were intact.

**Fig. 2:**
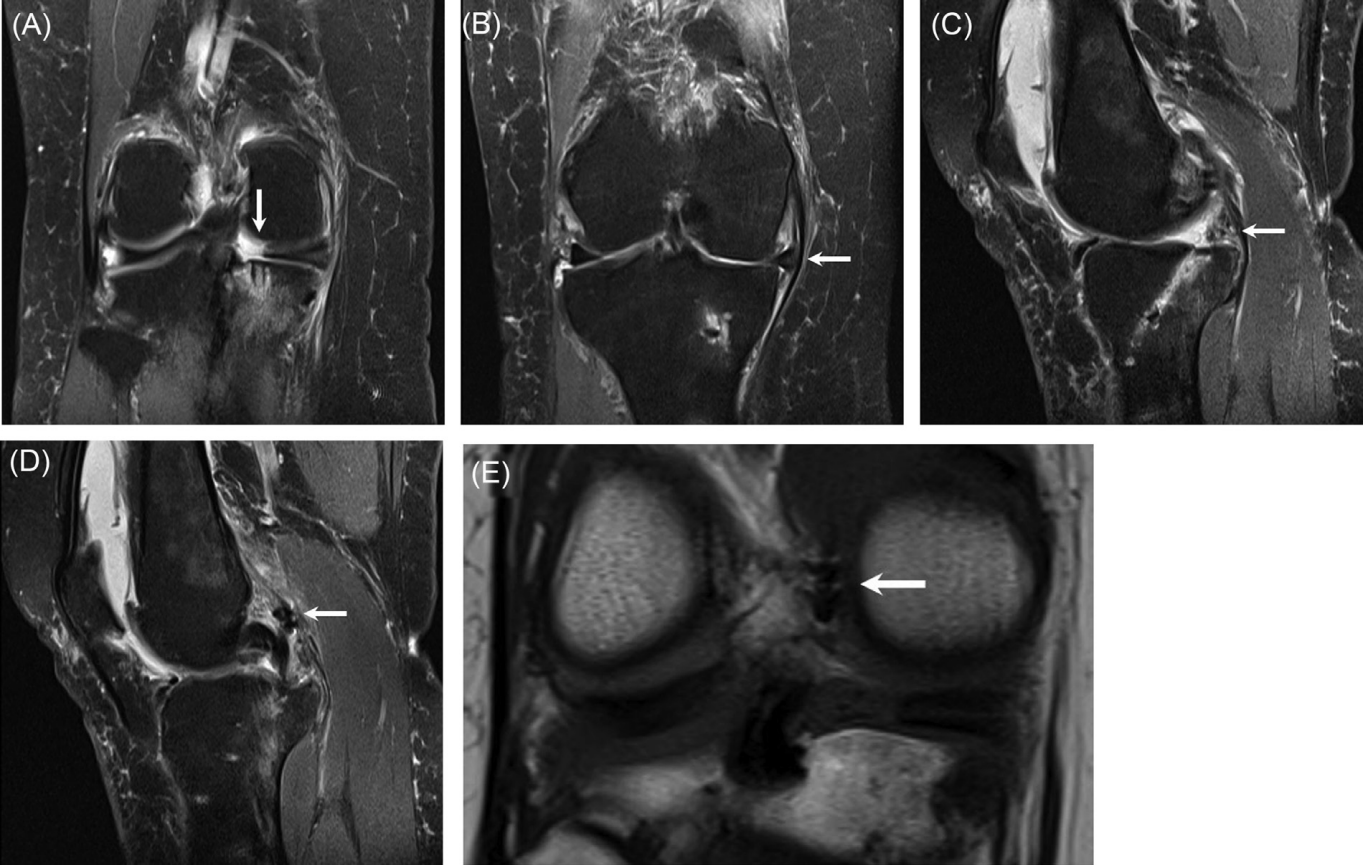
(A) Postoperative MRI. Coronal PD FS MRI image shows discontinuity of the posterior root of the medial meniscus at the level of the transtibial tunnel, consistent with tear (arrow). (B) Postoperative MRI. Coronal PD FS MRI image shows increased extrusion of the body of the medial meniscus from the joint space (compared to Fig. 1B), with bowing of the overlying medial collateral ligament (arrow). (C) Postoperative MRI. Sagittal PD FS MRI image shows abnormal signal and morphology of the posterior root of the medial meniscus (arrow) which does not appear continuous with the transtibial tunnel. The transtibial tunnel extends obliquely from posterosuperior to anteroinferior in the proximal tibia. (D) Postoperative MRI. Sagittal PD FS MRI image shows a focus of susceptibility artifact posterior to the intercondylar notch, likely representing a displaced suture (arrow). This is posterior to the posterior cruciate ligament. (E) Postoperative MRI. Coronal T1 MRI image shows a hypointense structure projecting in the posterior aspect of the intercondylar notch, likely representing a displaced suture (arrow).

Postoperatively, she was put in a TROM (total range of motion) brace locked in extension. Touchdown weightbearing was recommended and formal physical therapy was started. After 5 weeks, she was weaned off the knee brace and allowed to increase activities. At 7 weeks, she felt sudden onset of severe pain after turning in bed. Repeat MRI at 9 weeks showed findings concerning for suture pullout of the meniscal repair (Fig. 2).

Repeat arthroscopy performed 3 weeks after the second MRI showed previous sutures through the posterior horn/root tissue, but the sutures had failed within the tibial fixation. The sutures had pulled into the medial compartment through the previous tibial tunnel. The posterior root was partially scarred back to the tibia. The root repair sutures were loosened and then cut with an arthroscopic suture cutter. The sutures were then retrieved and removed using a grasper from the medial portal. Both repair sutures were removed from the knee. There was progressed grade IV chondrosis in the medial femoral condyle and grade II chondrosis in the medial tibial plateau.

The patient reported decreased pain postoperatively, without evidence of complications 6 weeks after the repeat arthroscopy.

## Case discussion

A meniscal root tear is a complete radial tear within 1 cm of the anterior or posterior tibial attachments of the menisci or an avulsion of the attachment [1]. The posterior root of the medial meniscus is more susceptible to tear due to reduced mobility and significant load burden [2]. Meniscal root tears can result in loss of hoop strength, meniscal extrusion, rapid cartilage loss, and subchondral insufficiency fracture [3]. There are multiple treatment options for meniscal root tears including conservative management, meniscectomy, and surgical repair [1,2,4]. Treatment selection is based on multiple factors including patient age, symptoms, degree of cartilage deterioration, and chronicity of the tear [1,2]. Surgical techniques include transtibial pullout, suture anchors, and side-to side-repair, and depend on the tear pattern [2]. Transtibial pullout repair is increasingly being used. Loosening of the transtibial pullout repair may occur if healing is incomplete prior to loading [5].

It is important for radiologists to recognize posterior meniscal root tears and be familiar with the normal and abnormal appearances of transtibial pullout repair [3,6]. We presented a case of a transtibial pullout repair complicated by suture pullout, recognized on MRI and confirmed on subsequent arthroscopy. This complication occurred approximately 7 weeks after the initial surgery without significant trauma, soon after the patient's knee brace had been discontinued. Fortunately, the posterior root had partially healed/scarred to its footprint by the time of subsequent arthroscopy and therefore re-repair was not required.

## Conclusion

Transtibial pullout repair is a relatively new surgical technique for treatment of meniscal root tears. It is important to confirm continuity of the posterior root all the way to the articular surface status post repair [3]. Lack of continuity of the posterior root with associated fluid signal should prompt search for displaced suture, progressive meniscal extrusion, cartilage loss, and subchondral insufficiency fracture, which are signs of failure of the repair.

## Patient consent

Written informed consent for the publication of this case report was obtained directly from the patient.
